# Deep Learning-Based Fatigue Monitoring in Natural Environments: Multi-Level Fatigue State Classification

**DOI:** 10.3390/bioengineering12121374

**Published:** 2025-12-18

**Authors:** Yuqi Wang, Ruochen Dang, Bingliang Hu, Quan Wang

**Affiliations:** 1 Key Laboratory of Spectral Imaging Technology, Xi’an Institute of Optics and Precision Mechanics (XIOPM), Chinese Academy of Sciences, Xi’an 710119, China; wangyuqi@opt.ac.cn (Y.W.);; 2 Key Laboratory of Biomedical Spectroscopy of Xi’an, Xi’an Institute of Optics and Precision Mechanics (XIOPM), Chinese Academy of Sciences, Xi’an 710119, China

**Keywords:** fatigue monitoring, machine learning, convolutional neuralnet work, electrocardiogram

## Abstract

In today’s fast-paced world, the escalating workloads faced by individuals have rendered fatigue a pressing concern that cannot be overlooked. Fatigue not only signals the need for individuals to take a break but also has far-reaching implications for both individuals and society across various domains, including health, safety, productivity, and the economy. While numerous prior studies have explored fatigue monitoring, many of them have been conducted within controlled experimental settings. These experiments typically require subjects to engage in specific tasks over extended periods to induce profound fatigue. However, there has been a limited focus on assessing daily fatigue in natural, real-world environments. To address this gap, this study introduces a daily fatigue monitoring system. We have developed a wearable device capable of capturing subjects’ ECG signals in their everyday lives. We recruited 12 subjects to participate in a 14-day fatigue monitoring experiment. Leveraging the acquired ECG data, we propose machine learning models based on manually extracted features as well as a deep learning model called C-BL to classify subjects’ fatigue levels into three categories: normal, slight fatigue, and fatigued. Our results demonstrate that the proposed end-to-end deep learning model outperforms other approaches with an accuracy rate of 83.3%, establishing its reliability for daily fatigue monitoring.

## 1. Introduction

### 1.1. Background

Fatigue is a complex and multidimensional phenomenon that lacks a standard or universal definition due to its association with a wide range of contributing factors [1]. Some scholars define fatigue as a physiological state characterized by “the consequences of prolonged mental or physical exertion that can impact an individual’s performance and diminish their mental alertness,” as articulated by the ‘Health and Safety Executive’ [2,3]. Another definition of fatigue describes it as “a sensation of tiredness, weariness, or drowsiness resulting from extended periods of mental and physical effort, prolonged periods of stress, exposure to adverse environmental conditions, or inadequate sleep.” Fatigue is a pervasive symptom, encountered not only in connection with acute and chronic illnesses but also in the context of normal, healthy functioning and daily life, often leading to reduced mental and physical capabilities [4,5].

Human fatigue can generally be categorized into two primary types: physiological fatigue and psychological fatigue, also known as mental fatigue [6,7]. Physical fatigue typically manifests as fatigue localized in specific muscle groups or as a general sense of bodily weariness. It encompasses systemic fatigue and local fatigue, the latter commonly stemming from excessive physical exertion. Physical fatigue is characterized by symptoms such as heightened muscle tension or poor local circulation, such as muscle fatigue and visual fatigue. Conversely, mental fatigue is primarily induced by elevated mental demands and intense cognitive activity, with some researchers employing the term “mental load” to describe it [8,9]. Brainworkers, such as researchers and scholars, are often susceptible to mental fatigue, which can lead to a decline in cognitive function, reduced attention span, and difficulties in high-level information processing. Individuals experiencing mental fatigue may also report feelings of drowsiness, a lack of energy, and challenges in maintaining focus. It is important to note that there is often an overlap between the causes of physical and mental fatigue in practice, as modern work and lifestyle habits frequently lead to a combination of both [10]. For instance, researchers engaged in extensive mental work may experience physical fatigue due to prolonged periods of sedentary activity and excessive eye strain [11].

Fatigue has far-reaching consequences that impact both individuals and society across various dimensions, including health, safety, productivity, and learning efficiency [12]. On an individual level, short-term fatigue can result in discomfort, reduced physical strength, and impaired motor control function. Additionally, individuals may experience a temporary decline in cognitive function, characterized by feelings of fatigue, diminished energy levels, and reduced attentiveness [13]. In rare cases, acute cognitive impairments may also occur [14,15].

Long-term fatigue poses more significant risks to individuals, potentially leading to various health issues, such as an increased prevalence of musculoskeletal disorders [16], chronic fatigue syndromes [17], and compromised immune function [18]. In China, there has been a steady rise in the number of individuals suffering from illness and sudden death attributed to prolonged overwork, mental stress, inadequate rest, and a lack of physical activity. This phenomenon, often referred to as “overwork death,” [19] has resulted in declining physical health and the development of chronic illnesses among intellectuals [8].

From a societal perspective, fatigue represents a significant contributing factor to fatal accidents involving operators and drivers, with potentially disastrous consequences [20]. Recent studies indicate that as much as 20% of fatal road accidents can be attributed to fatigue [21]. According to the National Highway Traffic Safety Administration, it is conservatively estimated that at least 100,000 vehicle accidents occur annually in the United States as a direct result of fatigued driving. These accidents lead to approximately 1550 deaths, 7100 injuries, and economic losses totaling USD120.5 billion [22]. Furthermore, the National Safety Council has found that 13% of workplace injuries can be directly linked to fatigue [23]. Research by Ricci has revealed that fatigued workers are less than half as productive as their well-rested counterparts, resulting in an annual financial loss exceeding $136 billion [24].

### 1.2. Related Work

Therefore, the detection of human fatigue assumes a pivotal role in mitigating the onset of diseases, injuries, accidents, and other detrimental consequences stemming from fatigue. To detect fatigue, researchers have devised numerous methods for measuring fatigue over the past few decades, which can be broadly categorized into two types: subjective measurements and objective measurements.

Subjective measurements involve the assessment of fatigue by having individuals rate their fatigue levels using various fatigue scales. Historically, many studies in the field of fatigue measurement have predominantly employed subjective methods. Researchers have developed a plethora of fatigue scales, and study participants provide responses to scale questions based on their personal subjective feelings. These scale responses are subsequently utilized to gauge their levels of fatigue [25]. Subjective methods have proven to be valuable for assessing the genuine subjective perception of cognitive fatigue and often serve as benchmark criteria for other fatigue measurement approaches. However, the use of fatigue scales necessitates that individuals set aside time to complete the scales, potentially causing interruptions during tasks. Consequently, these methods may not be suitable for unobtrusive tasks. Furthermore, subjective measurements lack the capacity to continuously and adaptively monitor fatigue in real-time. Moreover, in practical applications, subjective measurements are susceptible to human bias and suboptimal data collection methodologies. According to aviation research, between 70 and 80 percent of pilots inaccurately reported their fatigue levels when completing self-assessment fatigue scales [26].

Conversely, objective fatigue measurement methods can be subdivided into two primary categories: behavioral-based methods and physiological-based methods. Behavioral-based methods identify fatigue by examining the correlation between specific behaviors and fatigue levels. These methods involve the continuous monitoring of behavioral attributes during specific tasks, allowing for the continuous measurement of fatigue in a time-continuous manner.

However, it is important to note that detecting fatigue in diverse tasks necessitates the monitoring of entirely distinct sets of behaviors. Consequently, developing separate monitoring systems for each unique task becomes a requirement. As a result, these behavioral-based methods have limited applicability and tend to excel only in specific domains, such as the assessment of driving fatigue [27].

In contrast, physiological-based methods employ various physiological sensors to continuously monitor individuals and assess their fatigue levels by analyzing the acquired bio-signals. These methods offer the advantage of adaptive real-time fatigue monitoring by collecting pertinent physiological data. Additionally, they are versatile, capable of being applied across various activities and tasks. Moreover, physiological-based fatigue measurement techniques can provide early alerts to individuals in the initial stages of fatigue, enabling the prediction of fatigue onset [28].

Over the past few years, there has been significant advancement in wearable technology, enabling precise measurement of one or more physiological signals associated with fatigue. These devices have the capability to monitor and record individuals’ physical indicators in real-time, facilitating the quantification of fatigue levels. This progress has paved the way for the development of fatigue prediction systems utilizing the gathered physiological data [29].

Aryal et al. developed a fatigue measurement system utilizing skin temperature and electrocardiogram (ECG) data acquired from wearable devices. They calibrated the fatigue state using Borg’s Rating of Perceived Exertion (RPE) scale (6–20). Their approach involved the application of machine learning models for fatigue classification, with the Support Vector Machine (SVM) exhibiting the best performance, achieving an accuracy of 82% in binary classification [2]. Huang et al. employed a single-channel electrocardiogram device called “LaPatch” to capture ECG signals from participants. They extracted eight heart rate variability (HRV) indicators as features for their model, enabling the automatic detection of fatigue states. Prior to the experiment, subjects were required to complete the Chalder Fatigue Scale to report their fatigue levels.

The study utilized four different machine learning algorithms, with K-Nearest Neighbor (KNN) achieving the highest accuracy in binary classification at 75.5% [30]. Anwer et al. recruited 25 healthy individuals and had them perform a simulated manual material handling task to induce fatigue. They assessed the fatigue state based on mean heart rate (HR) and local skin temperature data obtained from the wearable device “EQ02 LifeMonitor.” Measurements were taken at baseline and after 30 min of the simulated fatigue task, revealing an increase in mean HR from 70 bpm to 120 bpm and a rise in local skin temperature from 31.5 °C to 34.9 °C during the task. Consequently, they argued that HR and skin temperature were significantly correlated with fatigue and could be employed as physiological indicators for assessing physical fatigue [31]. Lee et al. employed two wearable devices, “Zephyr Bioharness sensors” and “ActiGraph GT9X,” to gather data on heart rate (HR), HRV, and energy expenditure from six construction workers during roofing tasks. Fatigue levels were determined using a custom questionnaire. The results showed a significant increase in HR, HRV, and energy expenditure over five days of roofing work. The authors concluded that the assessment of HR, HRV, and energy expenditure could effectively identify physical fatigue [32].

Umer et al. conducted a study involving 10 subjects under experimental conditions to investigate their fatigue states. They recorded heart rate (HR) and skin temperature using the ‘EQ02 LifeMonitor’ during a simulated manual material handling task. To induce a consistent sensation of whole-body physical exertion, the experiment involved carrying a 15 kg box along a 14 m path with alternating cycles of with and without the box. Given that all participants were university students, this task proved physically challenging for them. Fatigue levels, evaluated using Borg’s RPE 6–20 scale, indicated that all participants reported high levels of exertion (scoring 15 to 20 on the RPE scale) during the task, signifying exhaustion after the experiment. They employed five supervised machine learning classifiers to assess fatigue, with the SVM model delivering the best performance, achieving an impressive accuracy of 95% [8].

Xiang et al. delved into the relationship between various physiological parameters (including electrodermal activity (EDA), respiration (RESP), skin temperature (SKT), R-R interval, and SDNN (standard deviation of NN intervals)) and fatigue. They simulated a construction working task to record the error rate in handling tasks and used this rate as a basis for determining the fatigue state. Their findings revealed a positive correlation between fatigue levels and electrodermal activity (EDA), respiration (RESP), and LF (low frequency)/HF (high frequency), while negative correlations were observed with the standard deviation of EDA, skin temperature (SKT), R-R interval, SDNN, and HFnorm [33].

Maman et al. recruited 8 participants for three in-lab experimental sessions, each involving a physically fatiguing task lasting three hours, divided into three one-hour periods of replicated tasks. Participants were equipped with four inertial measurement units (IMUs-Shimmer3) and one HR monitor to collect jerk data from acceleration and HR information. Logistic regression was used to detect the fatigue state, yielding favorable results [34]. Lamooki et al. enlisted 16 subjects and attached an IMU (Shimmer 3) to their right ankle. During the experiment, subjects were required to complete a 3 h physical task that included loading weighted cartons onto a dolly, pushing the dolly along a set path, and unloading the cartons at the starting location. They introduced a novel method that utilized sole acceleration data from the ankle to detect fatigue, eliminating the need for extensive data for algorithm calibration [35].

Karvekar et al. utilized accelerometers embedded in smartphones to collect motion parameters. Subjects performed fatiguing exercises like squatting and rated their perceived exertion using Borg’s RPE scale after each set of exercises. These ratings were used to label the gait data. They developed an SVM model to classify individuals’ fatigue states, achieving an accuracy of 61% [36]. Kushan and Krüger proposed an approach that collected IMU signals from the PowerGrasp exosuit and used acceleration and jerk metrics as inputs for their machine learning model. Subjects were tasked with consecutive assembly and disassembly cycles of a cover panel, with data recorded from IMUs on the upper arms and chest. After each cycle, fatigue levels were assessed based on the Borg CR10 scale. This study also employed an SVM model for binary classification of fatigue states, achieving a commendable accuracy of 83% [6].

Beyond ECG-based approaches, electroencephalogram (EEG) signals have emerged as another important modality for fatigue and drowsiness detection due to their ability to directly capture brain electrical activity associated with cognitive states. Recent advances in machine learning and deep learning have significantly enhanced EEG-based fatigue classification performance. Othmani et al. [37] conducted a comprehensive survey of EEG-based neural network approaches for fatigue and drowsiness detection, systematically investigating shallow and deep neural network methods and demonstrating that EEG is widely recognized as a dependable indicator for evaluating drowsiness, fatigue, and performance levels due to its robust association with these states. The effectiveness of frequency-domain features has been particularly notable, as evidenced by Adhikari et al. [38], who analyzed frequency domain features using variational mode decomposition (VMD) for EEG-based emotion classification, showing that extracting frequency domain features using sliding windows significantly enhances the efficiency of analyzing induced emotions. Furthermore, sophisticated feature engineering approaches have proven crucial for EEG-based fatigue detection. Tuncer et al. [39] proposed multilevel feature extraction using one-dimensional binary patterns combined with discrete wavelet transforms, along with iterative hybrid feature selection methods (ReliefF and iterative neighborhood component analysis), achieving 95.1% accuracy in driving fatigue detection. While EEG provides direct insight into cognitive states and mental fatigue, it typically requires more intrusive electrode placement on the scalp compared to ECG, making it less practical for continuous, long-term monitoring in natural daily environments. This motivates our focus on ECG-based approaches, which offer a favorable balance between measurement convenience, user compliance, and reliable fatigue assessment for everyday applications.

Photoplethysmography (PPG) is another commonly used physiological signal in wearable devices. PPG is an optical technique that measures blood volume changes through light absorption, providing information about heart rate and cardiovascular dynamics. While PPG has been explored for fatigue monitoring and can be combined with ECG for multimodal approaches, it is more susceptible to motion artifacts in natural daily environments compared to ECG.

### 1.3. Motivation and Contributions

Based on the studies discussed above, the implications of fatigue have raised significant concerns and sparked initial research efforts. In the realm of fatigue monitoring, the latest research trend involves integrating wearable devices with various sensors to achieve effective and personalized fatigue detection. However, it is worth noting that most previous studies have been conducted in controlled experimental settings. In these studies, subjects were required to complete specific tasks designed by researchers to induce relatively profound fatigue over extended periods of stimulation. These tasks often presented significant challenges to the subjects and were quite distinct from their daily activities. Consequently, heavy and prolonged tasks almost invariably resulted in deep fatigue among the participants.

Given this experimental context, the outcomes of these experiments were largely dependent on the extent of fatigue induced. Consequently, the majority of existing fatigue detection studies have primarily focused on inducing deep fatigue. While these studies have achieved accuracy, validity, and reliability in controlled settings, it remains uncertain whether these findings can be extrapolated to real-life scenarios characterized by substantially different environments. Unfortunately, the recognition of daily fatigue in free-living environments has received relatively less attention.

Detecting relatively mild fatigue without the need for specific stimulations holds significant meaning because it can offer a true reflection of an individual’s actual fatigue state in their daily life. Table 1 provides a summary of the key characteristics of previous research and highlights their variations and limitations.

The motivation of this study can be summarized as follows:

1. To accurately reflect and detect individuals’ actual fatigue states in daily life, we have developed a daily fatigue detection system designed for natural environments without the need for additional stimulating tasks, and assessed its feasibility.
2. To make the experiment as non-invasive and less burdensome as possible for participants, we have explored the viability and reliability of monitoring fatigue solely based on ECG signals.
3. We aim to evaluate the effectiveness of ECG signal-based multi-classification, including the detection of relatively mild fatigue commonly experienced in daily life.

The primary contributions of this study are as follows:

1. We have designed a flexible, low-power wearable device for measuring multiple physiological parameters and a monitoring system.
2. We conducted experiments with subjects in their natural everyday environment without additional stimulating tasks. Participants recorded their fatigue state using standard fatigue scales at specific times each day, and their ECG signals were collected using our self-developed wearable device.
3. We extracted fatigue-related ECG features and developed machine learning models to assess fatigue. To improve model performance and avoid time-consuming manual feature extraction, we also introduced a deep learning model for three-level fatigue assessment.

The overall structure of the study consists of five chapters, including this introductory section. Section 2.3 outlines the system structure of this study, including the design of the wearable device. Section 2.1.1 and Section 2.1.2 discuss the experiments, introducing the experimental setup and data collection process. Section 2.1.3, Section 2.2.1 and Section 2.2.2 cover ECG signal preprocessing, major feature extraction, and details of the machine learning and deep learning models proposed. Section 2.4, Section 3.1, Section 3.2 and Section 3.4 present the results achieved by our proposed method to demonstrate its effectiveness. Finally, the conclusion of this work and future directions are presented in Section 5.

## 2. Materials and Methods

### 2.1. Dataset and Experiments

#### 2.1.1. Experimental Procedure

The experimental design of this study aimed to capture ECG signals from subjects in entirely unaltered, real-world settings, free from any additional stimulating tasks. This approach allowed us to reflect real daily fatigue. A total of 12 subjects were recruited, all of whom were either students or staff members from the faculty of sports, comprising 7 males and 5 females. In addition to their academic and research responsibilities, these individuals engaged in daily sports training, a physically demanding activity that often resulted in physical fatigue. It is worth noting that all subjects had a clean bill of health with no history of heart disease. The subjects’ average age ranged from 21 to 32 years, and they ensured they obtained sufficient sleep each night throughout the experimental period, refraining from the consumption of alcohol or caffeine to prevent any physiological disruptions.

**Figure 1:**
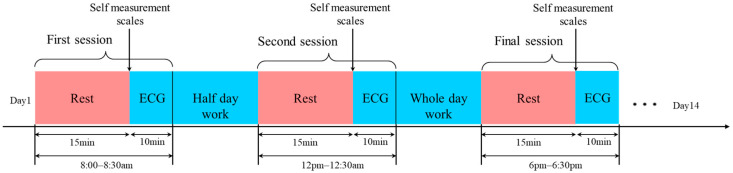
The flow chart of the whole experiment.

All participants provided written informed consent after receiving comprehensive information about the study procedures, data usage, and their right to withdraw at any time. To ensure data privacy and confidentiality, all collected physiological data were stored securely on local servers with access restricted to authorized project personnel only. The study was conducted in accordance with the Declaration of Helsinki, and the protocol was approved by the Ethics Committee of China-Japan Friendship Hospital (2021-7) on 21 July 2021. The experiment spanned a duration of two weeks, during which every subject continued to lead their normal lives and carry out their routine activities, including studying, sports training, and research. Typically, individuals tend to be non-fatigued in the morning, with fatigue levels typically increasing after the day’s work, as depicted in Figure 1, which illustrates the entire experiment process.

As depicted in Figure 1, the experiments were conducted on the same day, with subjects participating in repeated experiments three times daily. The first session took place from 8:00 a.m. to 8:30 a.m., right after subjects had just woken up, and they were typically in a non-fatigued state before commencing their daily activities. Following the completion of half-day tasks, subjects underwent the second session experiment from 12:00 p.m. to 12:30 p.m. The final experiment was scheduled for each evening between 6:00 p.m. and 6:30 p.m., a time when most subjects experienced a state of fatigue after a full day’s work.

During each session, subjects were initially instructed to remain at rest for fifteen minutes to minimize the influence of short-term activity on ECG signals. Prior to the commencement of ECG signal measurement, subjects were required to complete a fatigue self-assessment scale to gauge their current level of fatigue. The results obtained from these scales would subsequently serve as the labeling for the collected data. Following this assessment, ECG signals were recorded from the subjects over a duration of 10 min.

To ensure that subjects could provide accurate assessments of their fatigue levels, we conducted extensive research on methods for subjectively measuring physical fatigue, drawing from previous studies. Several studies have employed the Rating of Perceived Exertion (RPE) scale, commonly known as the Borg 6–20 scale, to capture subjects’ self-perceived levels of physical fatigue. Others have devised their own questionnaires to quantify physical fatigue or simply inquired about the presence of fatigue. While the questionnaires designed in previous research have been used exclusively for their respective studies, often lacking validation, simply asking about the presence of fatigue is non-scientific and insufficient for subjects to comprehensively evaluate their fatigue status.

In this study, subjects employed the Borg 6–20 scale to rate their physical fatigue levels, providing a validated and widely accepted method. The scale results were categorized into three levels based on scores, corresponding to three fatigue states: normal (not fatigued), slight fatigue, and fatigued. These results were subsequently used as labels for the ECG signals collected during the experiments.

**Figure 2:**
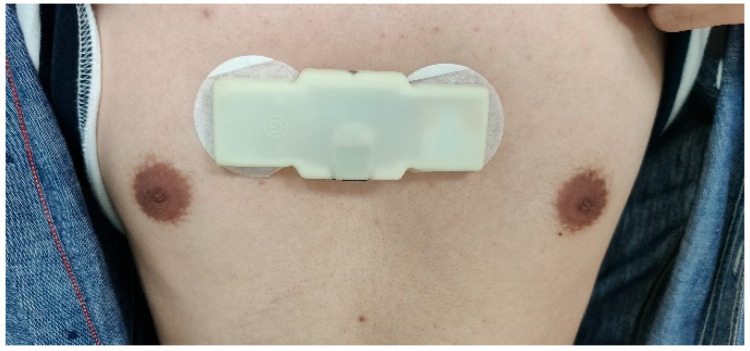
The wearing position of the device.

The developed wearable device was employed to measure the single-lead ECG signal of subjects using the chest attachment method. The electrodes were positioned at the sternum of the subjects, as depicted in Figure 2:

#### 2.1.2. Data Acquisition

The ECG signal was recorded at a sampling frequency of 200 Hz, and data communication was achieved through Bluetooth 4.0. As indicated in Figure 1, during each session, the ECG signal of subjects was acquired continuously for a period of 10 min. The 10 min ECG data were subsequently divided into a series of 30 s segments. This division meant that every 10 min ECG dataset was segmented into 20 individual segments. Segments originating from the same 10 min recording were assigned the same label, which could be “normal,” “slight fatigue,” or “fatigued,” based on the subjects’ self-assessment using the Borg 6–20 scale.

All the segments were further randomly divided into a training set and a testing set in an 8:2 ratio, ensuring the data was properly split for model training and evaluation.

Given that the experiments in this study were conducted in a non-experimental setting, it is natural that subjects may have encountered some challenges and made errors. These errors included issues such as improper device attachment, forgetting to activate the device, neglecting to change the electrode patch before the test, or failing to complete the self-measurement form before testing. Consequently, some of the collected data was rendered invalid. After excluding these invalid data points, a total of 2640 ECG data fragments were obtained from the experiments. This dataset consisted of 719 non-fatigue ECG fragments, 963 slight fatigue ECG fragments, and 958 fatigued ECG fragments.

To facilitate model training and evaluation, the data was divided based on an 8:2 ratio for the testing set, comprising 143 non-fatigue ECG fragments, 192 slight fatigue ECG fragments, and 191 fatigued ECG fragments.

#### 2.1.3. ECG Signal Pre-Processing

The initially acquired ECG signal was contaminated with noticeable industrial frequency interference and unavoidable baseline drift, necessitating preprocessing. To address these issues, we implemented a preprocessing approach.

The preprocessing approach addressed the following noise types commonly encountered in wearable ECG recordings: (1) Baseline wander: Low-frequency drift caused by respiration and body movements was removed using the comb filter described above. (2) Powerline interference: 50 Hz industrial frequency interference was eliminated using the 5-point sliding average filter as mentioned. (3) Muscle artifacts: High-frequency noise from muscle contractions was suppressed by applying a bandpass filter (0.5–40 Hz), which retained ECG signal components while removing muscle noise outside this range. (4) Electrode motion artifacts: Segments with severe motion artifacts were identified through visual inspection and automated amplitude thresholding, and excluded from subsequent analysis to ensure data quality.

**Figure 3:**
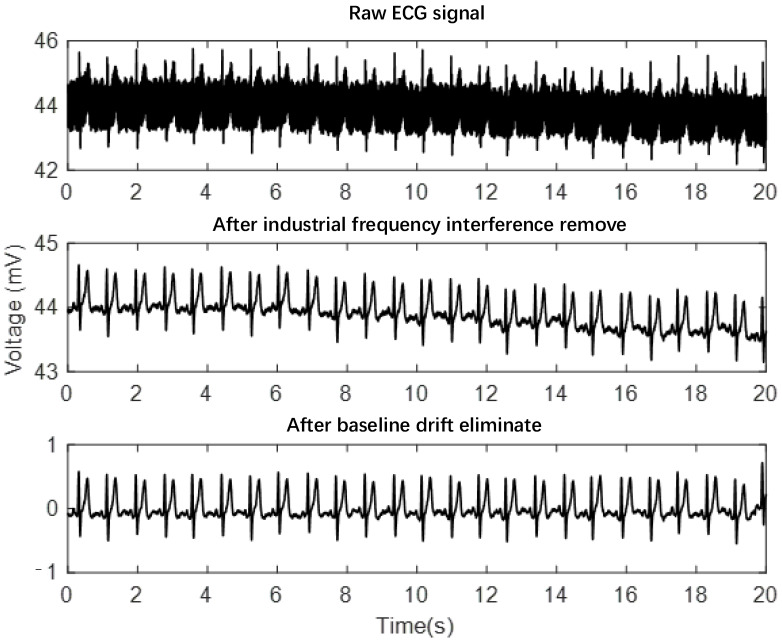
ECG signal before and after pre-processing.

As evident from Figure 3, the ECG signal becomes notably smoother and clearer after preprocessing. The preprocessing steps have led to a substantial enhancement in signal quality, resulting in a high signal-to-noise ratio.

### 2.2. Methods

#### 2.2.1. Machine Learning Methods

Conventional machine learning methods, which rely on manual feature extraction, were initially employed to classify fatigue states. Among these methods, heart rate (HR) stands out as the most commonly used physiological parameter for monitoring physical fatigue. The cardiovascular load increases as muscles contract during physical work, demanding more blood flow to the muscles. This results in a faster heart rate as the heart works to enhance blood transportation. Consequently, HR serves as a relatively objective indicator of physical fatigue [40].

Additionally, heart rate variability (HRV) is a classical approach to analyze ECG signals. HRV reflects minor variations in the intervals between adjacent heartbeats. The activity of the autonomic nervous system (ANS) is a reliable indicator closely correlated with human health [41]. ANS plays a pivotal role in cardiovascular function and is associated with various medical conditions, including fatigue states. The ANS comprises two components: the sympathetic nervous system (SNS) and the parasympathetic nervous system (PSNS) [42]. Fatigue can alter the balance and tension between these two systems, and HRV can quantitatively assess these changes, making it a valuable indicator of fatigue [43].

Therefore, in the context of fatigue research, HRV has been regarded as a robust metric.

To extract fatigue-related ECG features, various techniques are applied, including time domain analysis, frequency analysis, and nonlinear analysis. Time domain analysis of HRV is based on the statistical analysis of discrete R-R intervals [44].

**Figure 4:**
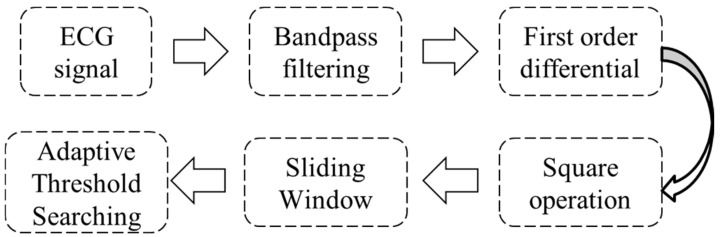
The flow chart of the P-T algorithm to extract Rpeaks.

**Figure 5:**
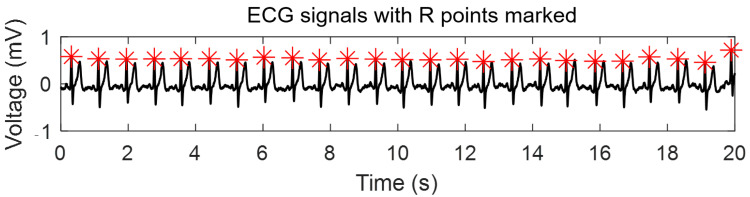
The ECG signal with R points marked.

In this study, the Pan-Tompkins algorithm, known for its strong performance in peak detection, was used to extract R peaks from the ECG signal. Subsequently, the RR interval was computed. The flowchart of the Pan-Tompkins algorithm for R peak extraction is depicted in Figure 4 below:

Following the completion of the aforementioned process, the R points in the ECG signal are identified, and the RR intervals are successfully extracted. Figure 5 visually represents the ECG signal with the R points marked.

Following the extraction of RR intervals, HRV features are computed based on this data. The detailed descriptions and calculations of these extracted features are provided in the Appendix A.

In total, 11 fatigue-related features are derived from the HRV analysis outlined above. These extracted features will serve as input to machine learning models designed to classify the fatigue state. In this study, three prominent machine learning models are developed as fatigue classifiers: Logistic Regression (Log Reg), Support Vector Machines (SVM), and Random Forest (RF). The classification performance of these models in the context of fatigue detection will be presented in the results section.

**Figure 6:**
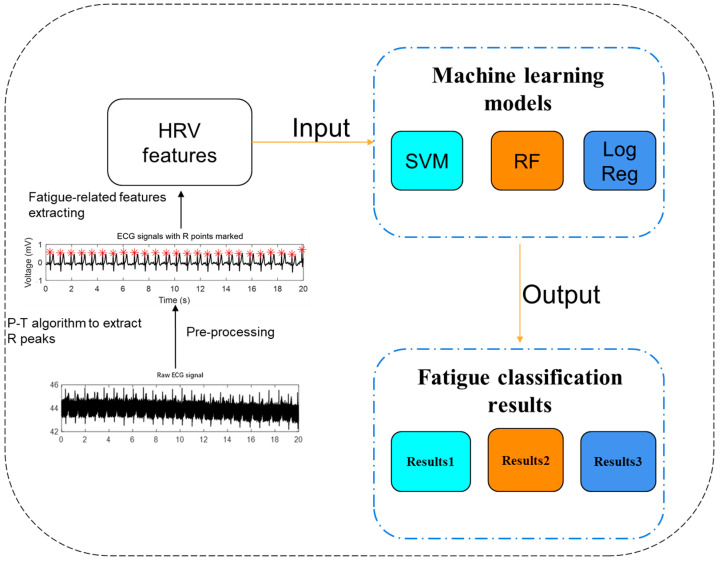
Process of fatigue detecting through manually extracted features-based machine learning methods.

The method flow for fatigue detection, involving manual feature extraction and machine learning, is summarized in Figure 6 below:

#### 2.2.2. The Proposed Deep Learning Method

Manually extracting features is a necessary step for traditional machine learning algorithms, but it can be time-consuming and may introduce instability in the quality of extracted features due to the subjective nature and experience of the human operator [20]. Furthermore, the performance of fatigue detection based on these models can be improved. To address these limitations, we have developed a deep learning model that eliminates the need for manual feature extraction from the ECG signal. This deep learning model operates in an end-to-end fashion, meaning that the fatigue recognition classification result can be directly obtained from the raw ECG signal using the proposed model.

Deep learning techniques have found widespread application in the field of medical health, and there is ample evidence to support the effectiveness of Convolutional Neural Networks (CNN) in analyzing ECG signals. Beyond medical applications, deep learning has demonstrated remarkable success in diverse classification tasks across multiple domains, including ground penetrating radar-based automated defect identification of bridge decks using hybrid approaches [45], structural damage detection in civil engineering, and automated quality inspection in manufacturing systems. These successful implementations of CNN-based classification in complex signal processing tasks inspired our approach to apply similar end-to-end deep learning architectures for ECG-based fatigue detection.

Consequently, our proposed model is built upon a CNN structure, allowing us to leverage the full potential of the raw ECG signal. CNN is a specialized multilayer neural network structure known for its ability to abstract raw inputs effectively. It possesses key characteristics such as local perception and weight sharing. Local perception means that each neuron in the convolution layer is only connected to neurons within a local window of the preceding layer. A typical CNN consists of a Convolution layer, a Pooling layer, and a Fully Connected (FC) layer. The convolution layer serves as the feature extractor in the CNN, and its operations can be represented as follows:

(1) $$x_{j}^{l}=f\left(\sum_{i\in M_{j}}\;x_{i}^{l-1}*k_{ij}^{l}+b_{j}^{l}\right)$$

$x_{j}^{l}$ represents the jth output of layer l, while $M_{j}$ denotes the set of inputs. $k_{ij}^{l}$ corresponds to the convolution kernel, and $b_{j}^{l}\;$ represents the adjustable bias term. The function $f\left(.\right)$ signifies the nonlinear activation function. In this study, we have adopted the Rectified Linear Unit (ReLU) as the activation function, which offers superior performance in mitigating the issue of vanishing gradients compared to traditional neural network activation functions, such as Sigmoid and Tanh. The ReLU is defined in Formula (2):

(2) $$\begin{array}{c} \mathrm{ReLU}\left(x\right)=\left\{\begin{array}{cc} x & x\geq 0\\ 0 & x<0 \end{array}\right.\\ =\max\left(0,x\right) \end{array}$$

**Figure 7:**
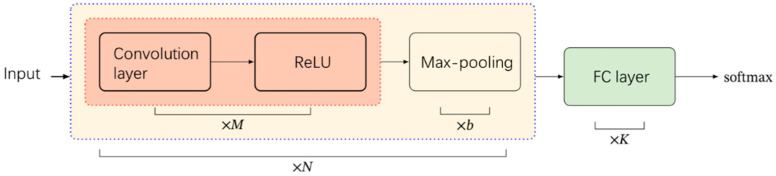
The basic framework of the first block.

After the convolution layer, the extracted feature map undergoes downsampling via the max-pooling layer. Additionally, the max-pooling layer contributes to the acquisition of a translation-invariant representation of the input [46]. Subsequently, a dropout layer with a probability of 0.5 is applied to reduce network complexity and mitigate the risk of model overfitting. The fundamental structure of the first block of the proposed model is illustrated in Figure 7:

The Table 2 shows the structure of the first block.

Up to this point, the first convolutional block has been assembled using the components we have discussed earlier: the convolution layer, the activation layer (ReLU), the pooling layer (Max-pooling), and the dropout layer with a probability of 0.5. When augmented with a Fully Connected (FC) layer, this first block constitutes a complete classical CNN model. However, CNN models are specialized for processing data with spatial features like images and do not inherently possess the capability to handle time series data or utilize their temporal information.

**Figure 8:**
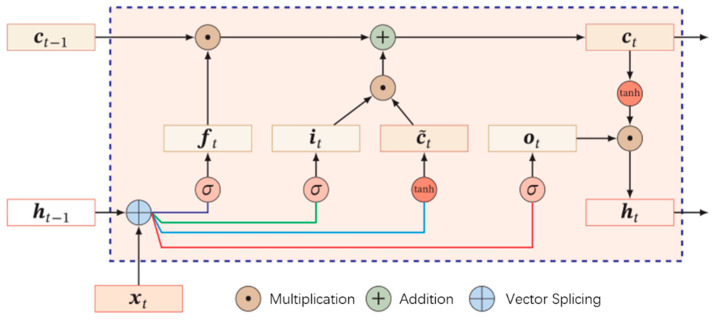
The structure of LSTM.

The physiological basis for fatigue monitoring in this study is the ECG signal, a quintessential time series data. The ECG signal at a given moment is intricately linked to its preceding and subsequent moments, reflecting temporal dependencies. To harness the deeper features within the ECG signal and further enhance the model’s performance for fatigue monitoring, we have devised a block based on Bidirectional Long Short-Term Memory (BiLSTM). LSTM, an improved variant of Recurrent Neural Networks (RNN), effectively addresses the issues of gradient explosion or vanishing that plague simpler RNNs. Figure 8 provides an overview of the LSTM structure.

The variable $c_{t}$ represents the internal state of the LSTM, which plays a crucial role in transferring linear cyclic information and output information to the external state $\;h_{t}$ in a nonlinear manner. The calculation process is as follows:

(3) $$\begin{array}{c} c_{t}=f_{t}⊙c_{t-1}+i_{t}⊙{\tilde{c}}_{t}\\ h_{t}=o_{t}⊙\tanh\left(c_{t}\right) \end{array}$$

The three gates, namely $f_{t}$ (Forget gate), $i_{t}$ (Input gate), and $o_{t}$ (Output gate), serve as control mechanisms for regulating the flow of information within the LSTM. They determine how information is passed between time steps. $c_{t-1}$ represents the memory unit from the previous time step, and ${\tilde{c}}_{t}$ is the candidate state computed through a nonlinear function, as calculated using Formula (4).

(4) $${\tilde{c}}_{t}=\tanh\left(W_{c}x_{t}+U_{c}h_{t-1}+b_{c}\right)$$

$f_{t}$ (Forget gate) controls how much information needs to be forgotten about the internal state of the previous moment $c_{t-1}$. $i_{t}$ (Input gate) then decides how much information needs to be kept about the candidate state ${\tilde{c}}_{t}$. While $o_{t}$ (Output gate) finally controls $c_{t}$ how much information needs to be outputted to the external state $h_{t}$ at the current time. The three gates in LSTM are all ‘soft gates’ with a value between (0,1). The calculation method for the three gates is:

The operation of the three gates in LSTM is as follows:

- $f_{t}$ (Forget gate) determines the extent to which information from the internal state of the previous moment $c_{t-1}\;$ should be forgotten.
- $i_{t}$ (Input gate) then decides how much of the candidate state ${\tilde{c}}_{t}$ should be incorporated and retained.
- $o_{t}$ (Output gate) controls how much of the information in $c_{t}$ should be outputted to the external state $h_{t}$ at the current time.

It is important to note that these three gates in LSTM are considered ‘soft gates,’ meaning they produce values between 0 and 1, thereby modulating the flow of infor-mation. The calculation method for these gates is determined by specific equations:

(5) $$\begin{array}{c} i_{t}=\sigma \left(W_{i}x_{t}+U_{i}h_{t-1}+b_{i}\right)\\ f_{t}=\sigma \left(W_{f}x_{t}+U_{f}h_{t-1}+b_{f}\right)\\ o_{t}=\sigma \left(W_{0}x_{t}+U_{o}h_{t-1}+b_{o}\right) \end{array}$$

The entire network has been designed to capture long-range temporal dependencies, and the operations described in Formulas (3)–(5) can be concisely summarized as follows:

(6) $$\begin{array}{c} \left[\begin{array}{c} {\tilde{c}}_{t}\\ o_{t}\\ i_{t}\\ f_{t} \end{array}\right]=\left[\begin{array}{c} \tanh\\ \sigma \\ \sigma \\ \sigma \end{array}\right]\left(W\left[\begin{array}{c} x_{t}\\ h_{t-1} \end{array}\right]+b\right),\\ c_{t}=f_{t}⊙c_{t-1}+i_{t}⊙{\tilde{c}}_{t},\\ h_{t}=o_{t}⊙\tanh\left(c_{t}\right) \end{array}$$

**Figure 9:**
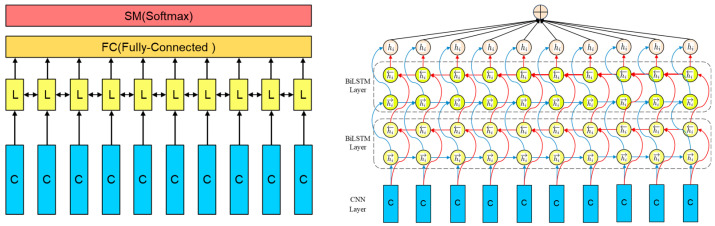
The structure and the data processing of the proposed CNN-BiLSTM model.

**Figure 10:**
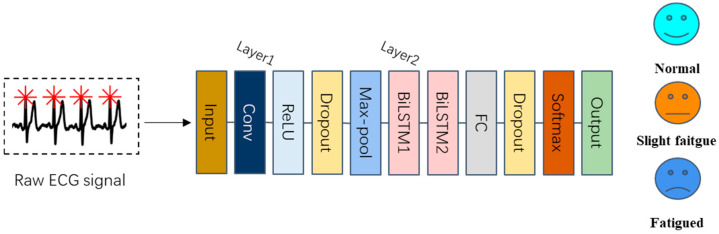
The structure of the proposed model.

BiLSTM, short for Bidirectional Long Short-Term Memory, represents an extension of LSTM and comprises two LSTM modules. These two LSTM modules share the same inputs, differing only in the direction of information transfer. In the context of the ECG signal, the output at a given time point depends not only on past information but also on future information. The BiLSTM module has the capability to extract features independently from both the forward and backward sequences and subsequently integrates them to determine the output. Figure 9 provides an overview of the BiLSTM structure:

The first block of our proposed model is succeeded by two BiLSTM layers, which delve deeper into the feature maps extracted by the initial block. These are then followed by the Fully Connected (FC) layer and the Softmax layer, together constituting the second block of the proposed model. Table 3 provides an overview of the structure of this second block:

The data processing pipeline for the proposed CNN-BiLSTM model is depicted in Figure 9:

By incorporating the BiLSTM block, our proposed model gains the capability to extract time series-related features from ECG signals and leverage their temporal information, thereby enhancing its performance. The comprehensive framework of the proposed model is illustrated in Figure 10:

To optimize the model’s performance, an array of experiments was conducted to fine-tune the hyperparameters. After a thorough exploration, the hyperparameters were ultimately determined as listed in Table 4:

### 2.3. Proposed System

We have developed a fatigue detection system designed to collect, store, transform, and analyze ECG signal data. This system comprises a wearable multi-physiological parameter measurement device, paired with a mobile application that facilitates data storage, real-time signal display, and monitoring. The primary achievements in the development of the wearable fatigue physiological parameter monitoring sensor encompass the device’s design, performance testing, and calibration against medical standard equipment.

#### 2.3.1. The Design of the Wearable Device

**Figure 11:**
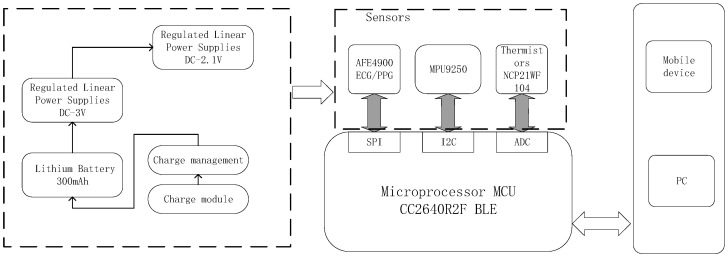
Block diagram of the fatigue monitoring system.

The overall architecture of the fatigue monitoring wearable device is illustrated in Figure 11:

As depicted in Figure 11, the fatigue monitoring system comprises two main components: the wearable device terminal responsible for data collection and the platform-side component for real-time signal storage, visualization, data processing, and analysis. The device terminal includes the power management module, signal acquisition module, and MCU master control module.

Serving as the central controller for this device, the CC2640R2F (Texas Instruments, Dallas, TX, USA) from TI is a wireless microcontroller (MCU) commonly utilized in low-power applications involving Bluetooth 4.2 and Bluetooth 5. The CC2640 boasts a 32-bit ARM Cortex-M3 core operating at 48 MHz, a 2.4 GHz RF transceiver antenna, and 128 KB of in-system programmable flash memory. ECG signal acquisition is facilitated by the ultra-low power integrated analog front-end AFE4900 (Texas Instruments, Dallas, TX, USA) from TI. The AFE4900 supports the simultaneous acquisition of one-channel ECG and three-channel photoplethysmography (PPG) signals. In this system, we store ECG and PPG signals in a 128-sample first-in-first-out (FIFO) queue block, with data retrieval performed through the Serial Peripheral Interface (SPI) interface.

**Figure 12:**
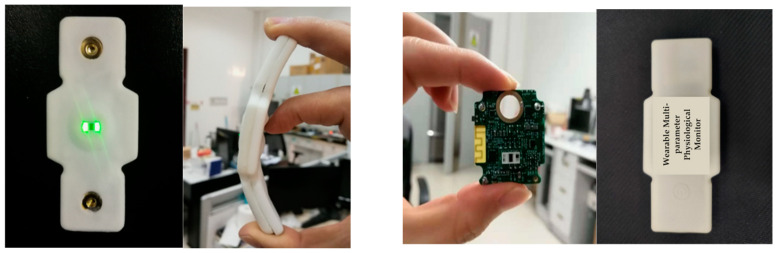
The physical maps of the developed wearable device.

**Figure 13:**
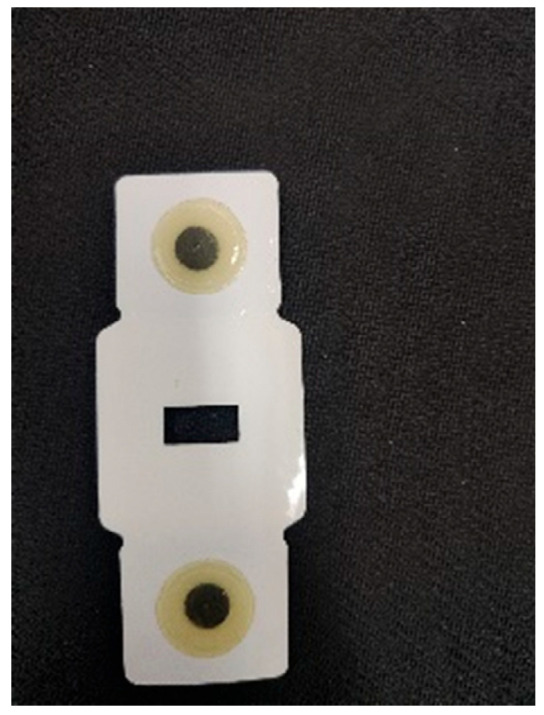
Ecg electrodes.

**Figure 14:**
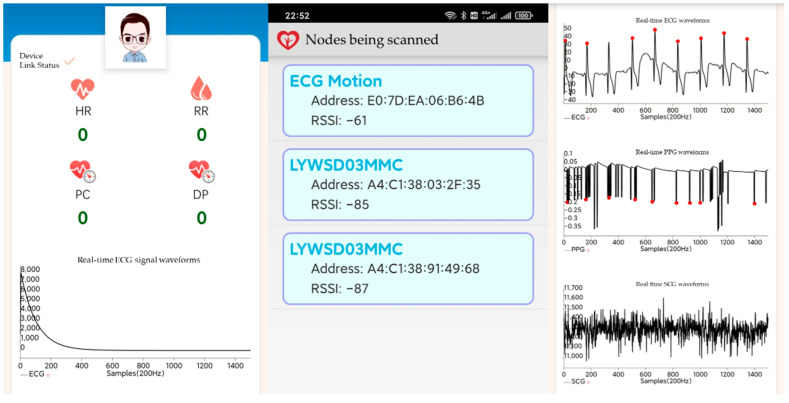
The pages of the physiological parameters system application.

The developed device also has the capability to measure photoplethysmography (PPG) signals, acceleration signals, and oxygen signals. However, for the purposes of this study, we exclusively utilized the ECG signal for fatigue detection. Therefore, we will not delve further into the design of acquisition modules for the other signals. The physical representations of the developed device are displayed in Figure 12 below:

The ECG electrodes have been customized to fit the dimensions of the developed device, as illustrated in Figure 13:

In this system, we have developed a corresponding application. The ECG signal acquired by the wearable device is transmitted to the mobile device via Bluetooth and can be viewed in real-time on the mobile device through the developed application. Figure 14 presents the application’s interface along with screenshots both before and after the commencement of measurement.

#### 2.3.2. The Performance Testing of the System

To ensure the reliability of the signal source and the preciseness of this study, the developed wearable device has been tested for performance by comparing and calibrating it with standard medical equipment. In this study, the standard medical monitor device BeneView T5 from Myriad was used to evaluate and calibrate the developed device. Six subjects with an average age of 26 participated in the test work. In the test, both the standard medical equipment and the developed device were used on subjects, the acquired signals were stored on a PC and then compared and analyzed.

**Figure 15:**
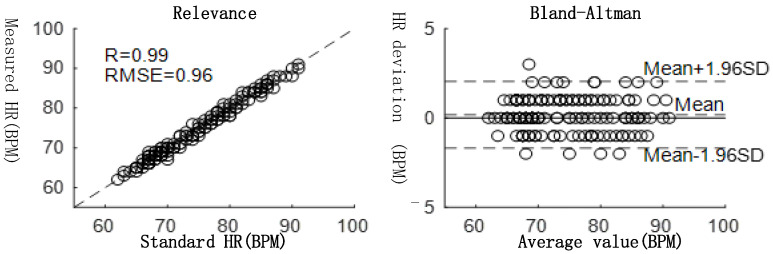
The developed device performance test results.

The evaluating indicators are the pulse rate scatter plots and Bland–Altman consistency analysis of heart rate which are shown in Figure 15.

As illustrated in Figure 15, the developed device exhibits a high level of compatibility with the standard medical monitor Myriad BeneView T5. The correlation coefficient between the measured heart rate parameters and the standard heart rate parameters was 0.99, and the root mean square error was 0.96 BPM.

### 2.4. Model Validation Metrics

The acquired dataset was employed to validate the proposed model. The segments from the dataset were further partitioned into training and test sets. Specifically, 80% of the dataset was randomly allocated to the training set, while the remaining 20% was designated as the test set. Initially, the proposed model was trained using the training set, and subsequently, it was evaluated on the test set for fatigue classification.

Moreover, to mitigate the risk of overfitting and assess the robustness of the proposed model, a 10-fold cross-validation was performed.

The classification metrics used for evaluation encompass accuracy, F1 score, Recall and precision, each of which is detailed as follows:

(7) $$Accuracy=\frac{TP+TN}{TP+TN+FP+FN}$$

(8) $$Precision=\frac{TP}{TP+FP}$$

(9) $$Recall=\frac{TP}{TP+FN}$$

(10) $$F1\;Score=\frac{2\times Precision\times Recall}{Precision+Recall}$$

TP (True Positives) means the values that are positive and predicted as positive. TN (True Negative) stands for values that are negative and predicted to be negative. FP (False Positives) denotes cases that are positive but classified as negative. FN (False Negative) is the value that are positive but classified as negative. All the results are the average of three classifications of fatigue (normal, slight fatigued, and fatigued).

TP (True Positives) refers to values that are genuinely positive and correctly predicted as such. TN (True Negatives) corresponds to values that are truly negative and accurately predicted as negative. FP (False Positives) represents instances where values are actually positive but have been erroneously classified as negative. FN (False Negatives) encompasses situations where values are indeed positive but have been incorrectly classified as negative. It is important to note that all reported results are the averages derived from three classifications of fatigue, including normal, slight fatigue, and fatigued states.

## 3. Results

### 3.1. Performance Results of Machine Learning Methods

As described in Section 2.2, our initial approach involved proposing conventional machine learning methods based on manual feature extraction for classifying the fatigue state. Specifically, we extracted a total of 11 fatigue-related HRV features from various domains, including time, frequency, and nonlinear (as detailed in Table A1). These features were used as input for our models.

Subsequently, we developed three widely recognized machine learning models to classify fatigue states: Logistic Regression (Log Reg), Support Vector Machines (SVM), and Random Forest (RF). The results of these classification efforts are presented in Table 5:

Table 5 reveals that among the three conventional machine learning models, the SVM model outperforms the others, achieving the highest accuracy at 0.643.

### 3.2. Performance Results of the Proposed Deep Learning Model

**Figure 16:**
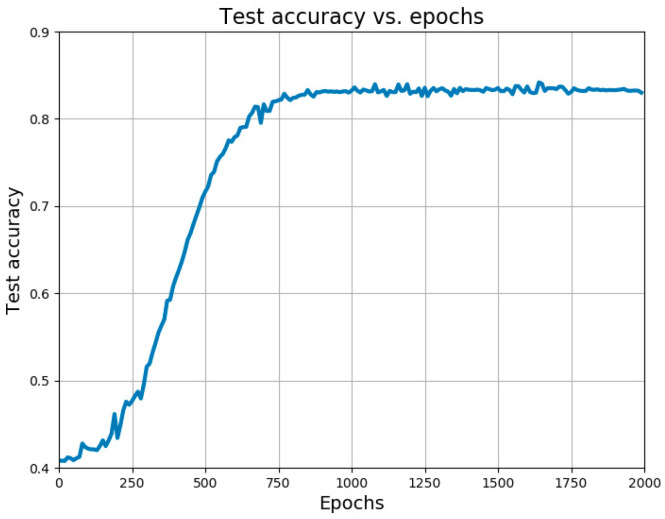
Model accuracy curve with epochs.

Despite the manual extraction of 11 features for fatigue classification, the best-performing model still achieved an accuracy of only 0.643, indicating room for improvement. Additionally, the process of manually extracting these features is time-consuming. Consequently, we have extended our efforts to develop a deep learning model that operates in an end-to-end fashion, as illustrated in Figure 16. The performance results of this proposed model are presented in Figure 16:

From Figure 16, it is evident that the proposed model gradually converges from epoch 0 to epoch 1000, after which the accuracy steadily improves. Ultimately, the accuracy stabilizes at 0.833. The precision and F1 score of this model are recorded as 0.847 and 0.822, respectively.

In comparison to the conventional machine learning methods that rely on manually extracted features, the proposed model demonstrates significant enhancements in the classification performance for fatigue states. Notably, the triple classification accuracy (comprising normal, slight fatigued, and fatigued states) achieved by the proposed model is nearly equivalent to the binary classification (non-fatigued or fatigued) reported in previous studies.

**Figure 17:**
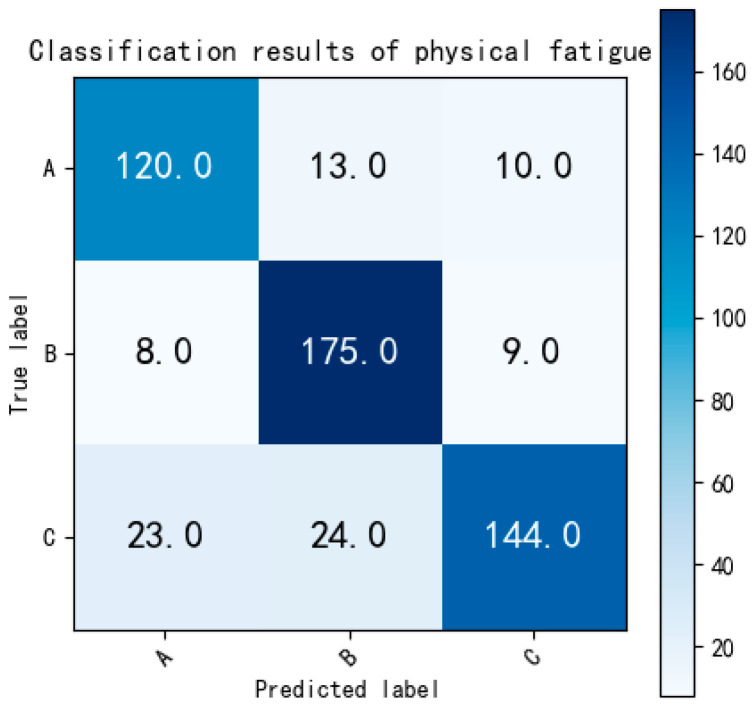
The confusion matrix of three-class classification of C-BL model. Labels: A = non-fatigue, B = slight fatigue, C = fatigued.

Figure 17 is the confusion matrix of three-class classification of C-BL model.

**Figure 18:**
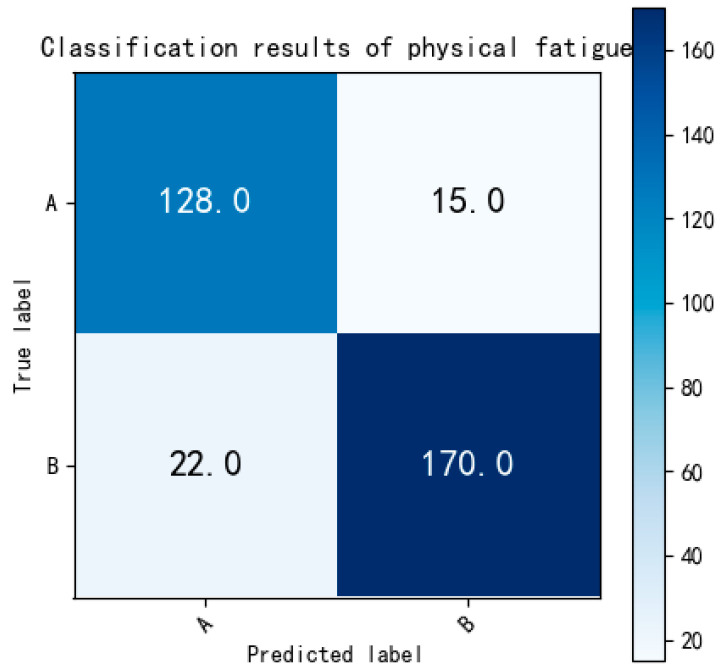
The confusion matrix of binary classification (non-fatigue and slight fatigue). Labels: A = non-fatigue, B = slight fatigue.

**Figure 19:**
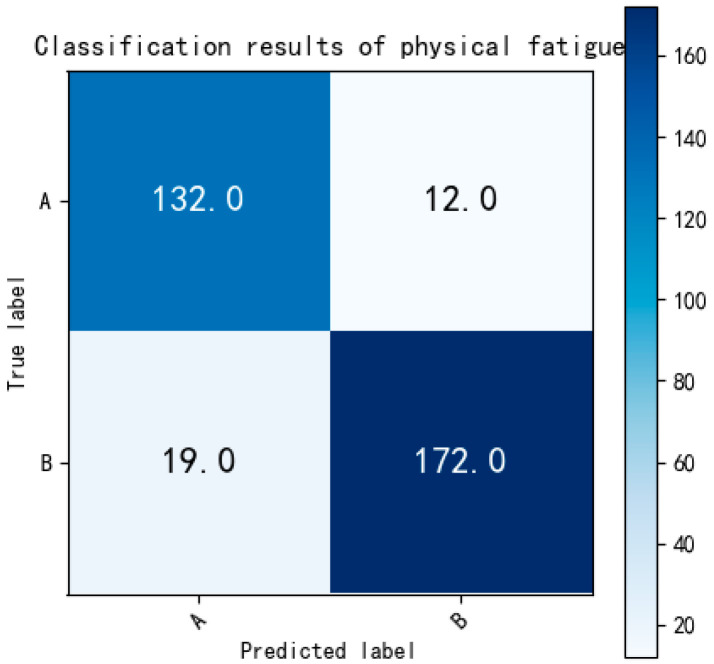
The confusion matrix of binary classification (non-fatigue and fatigued). Labels: A = non-fatigue, B = fatigued.

**Figure 20:**
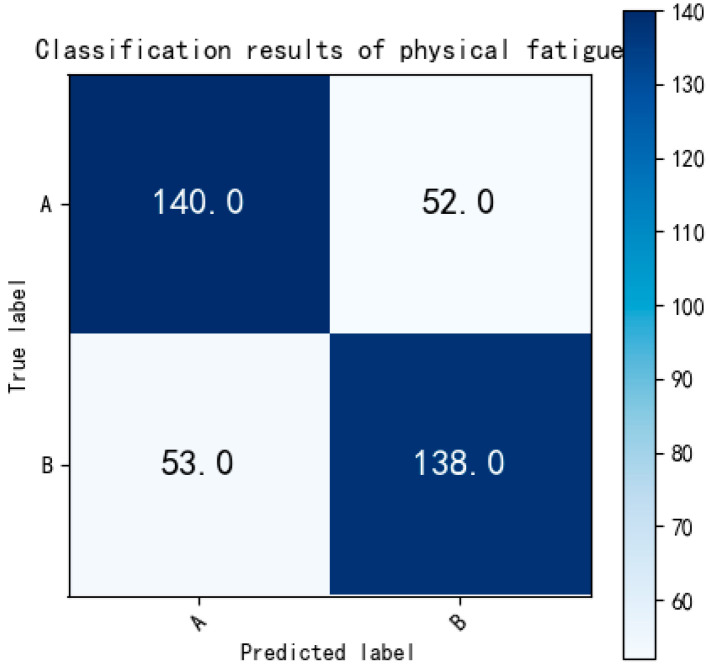
The confusion matrix of binary classification (slight fatigue and fatigued). Labels: A = slight fatigue, B = fatigued.

In order to conduct a more detailed evaluation of the classification performance for various levels of physical fatigue, the data pertaining to three different fatigue levels were separately considered as binary classification tasks. The testing set for these tasks comprises 143 ECG fragments for non-fatigue, 192 ECG fragments for slight fatigue, and 191 ECG fragments for fatigued states. To begin, we initially focused on classifying ECG data between non-fatigue and slight fatigue states. The outcomes of this classification are depicted in Figure 18:

Figure 18 indicates that a total of 128 non-fatigue and 170 slight fatigue ECG fragments were accurately classified. The binary classification accuracy between non-fatigue and slight fatigue is reported as 88.96%, surpassing the accuracy achieved in the multi-class classification task (83%). Moving on to the classification between non-fatigue and fatigued states, the results are presented in Figure 19:

As Figure 19 shows, there were 132 non-fatigue and 172 fatigued ECG fragments correctly classified. The binary classification accuracy of non-fatigue and fatigued is 90.75%. Finally, the slight fatigue and fatigued state were evaluated and the result is shown in Figure 20:

Figure 20 indicates that a total of 140 slight fatigue and 138 fatigued ECG fragments were correctly classified. The binary classification accuracy between the slight fatigue and fatigued states is reported as 72.58%.

Summarizing the results from Figure 17 to Figure 20, it becomes evident that the non-fatigue state can be more readily distinguished from the other two states (slight fatigue and fatigued). However, differentiating between the states of slight fatigue and fatigued proves to be more challenging, as the binary classification accuracy is lower than that of the multi-classification task.

### 3.3. Overall Performance Comparison

To establish a comprehensive performance hierarchy, we evaluated our CNN-BiLSTM model against baselines ranging from the simplest threshold-based methods to sophisticated deep learning approaches.

Simple Baseline Methods: We implemented three categories of interpretable baseline classifiers: (1) Single-feature threshold classification using optimal thresholds for each of the 11 HRV features individually, with RMSSD achieving the best accuracy of 54.8%; (2) Rule-based classification using hand-crafted decision rules combining RMSSD and Mean RR intervals (57.2%); and (3) Weighted scoring system combining three key HRV features with equal weights (59.6%). Results (Table 5) demonstrate a clear performance hierarchy.

Simple threshold and rule-based methods achieve 54.8–59.6% accuracy, providing perfect interpretability but insufficient reliability for clinical use. Traditional machine learning methods (SVM, RF, Logistic Regression) reach 64.3–72.1%, representing substantial improvements over simple baselines. Deep learning approaches further advance performance, with CNN-only and BiLSTM-only achieving 72.05% and 75.86%, respectively. Our proposed CNN-BiLSTM model achieves 83.3%, representing a 23.7 percentage point improvement over the best simple baseline and a 19.0 point improvement over the best traditional ML method.

This comprehensive comparison clarifies the interpretability-performance trade-off: while simple methods offer transparency and require no training, their limited accuracy renders them unsuitable for reliable fatigue monitoring. The substantial performance gain of our deep learning approach justifies the increased complexity for safety-critical applications. The Table 6 shows the comprehensive performance.

### 3.4. Performance Results of Ablation Experiments

As detailed in the Methods Section, the proposed model is a composite of multiple modules, comprising a CNN layer (Layer 1) and an LSTM layer (Layer 2). To gain insights into the individual contributions of each technique to the final performance, ablation experiments have been conducted.

**Figure 21:**
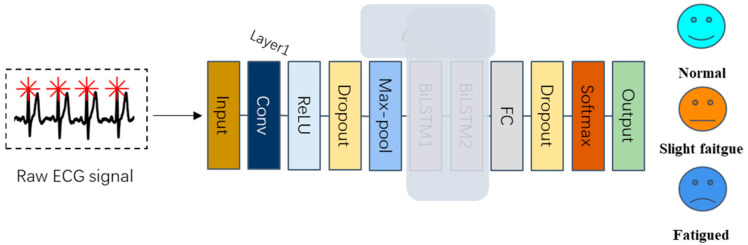
The structure of the proposed model without layer 2.

**Figure 22:**
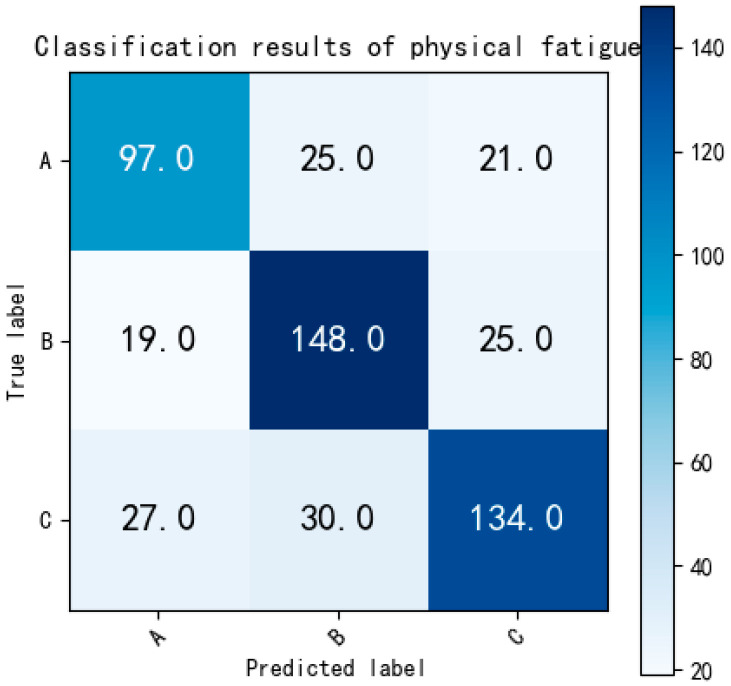
The confusion matrix of the three-class classification of CNN, Labels: A = non-fatigue, B = slight fatigue, C = fatigued.

To begin, we assessed the contribution of the BiLSTM layer (Layer 2). The structure of the model after removing the BiLSTM layer is depicted in Figure 21:

Without layer 2, the model lost the memory function. The performance of the CNN model is shown in Figure 22:

As illustrated in Figure 22, the removal of the BiLSTM layer has a notable detrimental effect on the model’s performance in fatigue state classification. The accuracy for multi-classification drops to 72.05%.

**Figure 23:**
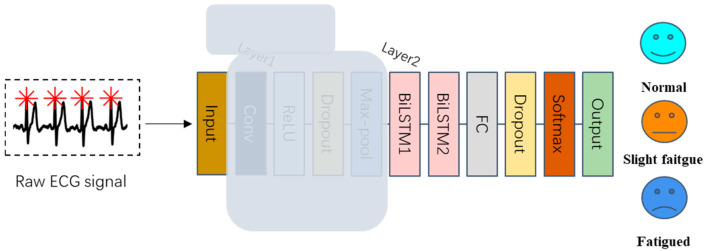
The structure of the proposed model without layer 1.

**Figure 24:**
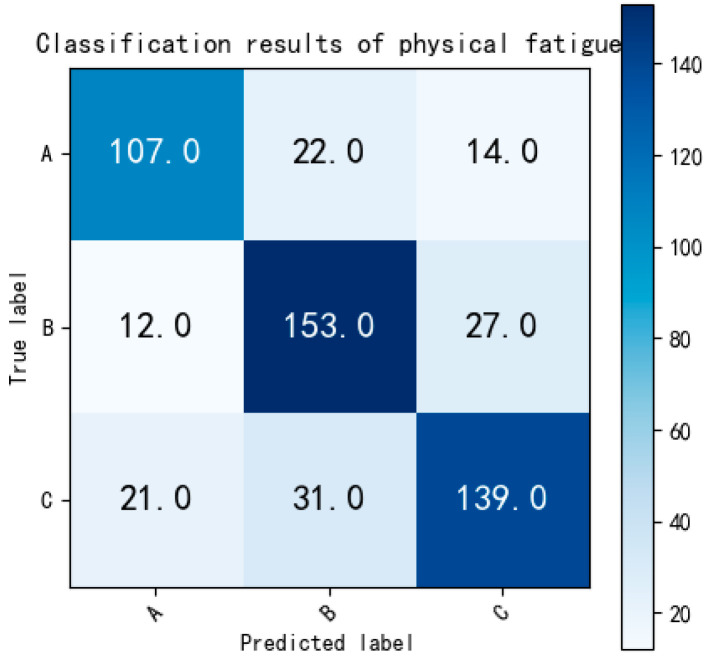
The confusion matrix of the three-class classification of BiLSTM, Labels: A = non-fatigue, B = slight fatigue, C = fatigued.

Next, we investigated the contribution of the CNN layer (Layer 1). The structure of the model after removing the CNN layer is presented in Figure 23:

The classification result of the model without the CNN layer (BiLSTM-only) is displayed in Figure 24:

As indicated by Figure 24, the removal of the CNN layer results in a decrease in the performance of the BiLSTM model in fatigue state classification. The accuracy for multi-classification is reported as 75.86%, which surpasses that of the single CNN structure model.

In light of these results, it can be concluded that both the CNN layer and the BiLSTM layer play pivotal roles in enhancing the performance of the CB-L model. The absence of either of these modules would significantly diminish the classification accuracy of the model.

### 3.5. Statistical Significance Testing

To rigorously validate performance improvements, we conducted McNemar’s tests comparing CNN-BiLSTM (C-BL) against all baseline models using paired predictions on the same test set. McNemar’s test is appropriate for comparing two classifiers on identical datasets. Results (Table 7) demonstrate that C-BL significantly outperforms all baselines with *p* < 0.01 for all comparisons. Against traditional machine learning methods, C-BL achieves improvements of 19.0–25.9% with highly significant *p*-values (*p* < 0.001), confirming substantial and statistically reliable gains. Ablation studies show C-BL significantly outperforms CNN-only by 11.25% (*p* = 0.0031) and BiLSTM-only by 7.44% (*p* = 0.0083), validating that both architectural components contribute significantly to overall performance. These statistical tests confirm that the observed improvements represent genuine advances rather than random variation.

### 3.6. Model Interpretability Analysis

To enhance model transparency for clinical applications, we implemented Gradient-weighted Class Activation Mapping (Grad-CAM) to visualize which ECG regions the CNN-BiLSTM model focuses on when making fatigue classification decisions.

**Figure 25:**
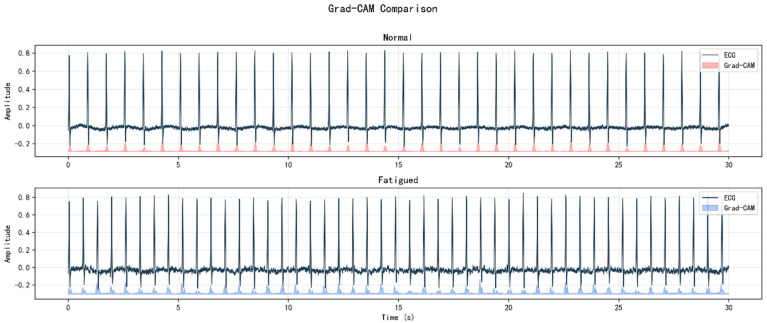

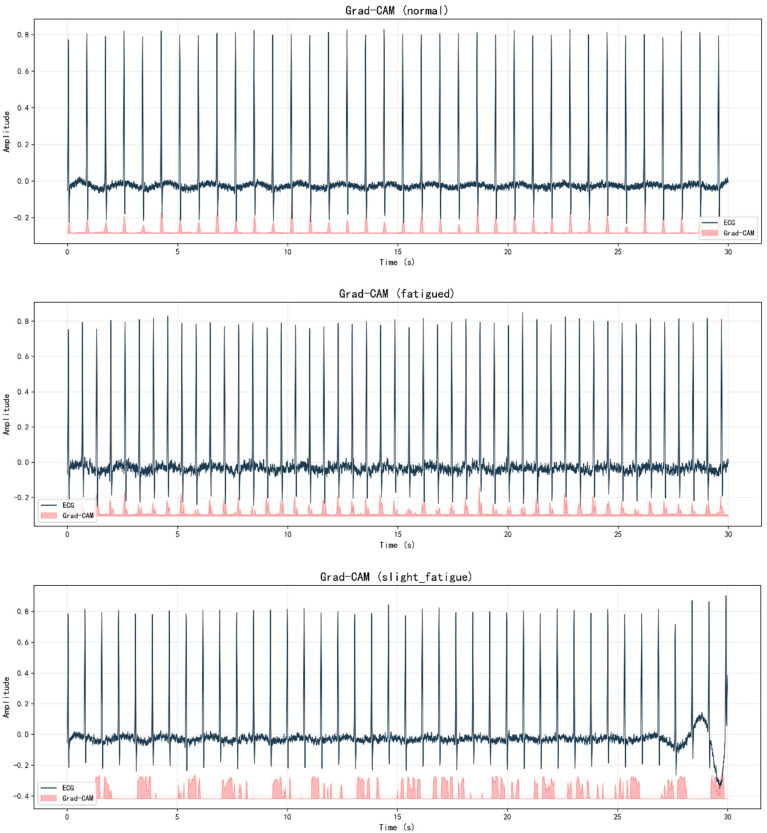
Grad-CAM Visualization.

Quantitative analysis across the three fatigue states reveals a progressive expansion of the model’s attention regions (Figure 25):

- Normal state: 13.32% activation area, primarily focusing on R-peak regions—Slight fatigue: 15.67% activation area (+17.6% vs. normal), expanding to RR intervals and T-wave morphology.
- Fatigued state: 16.46% activation area (+23.5% vs. normal), requiring comprehensive analysis of the entire cardiac cycle.

This progressive expansion aligns well with cardiovascular physiology principles. During fatigue, autonomic nervous system regulation becomes less stable, leading to increased heart rate variability and alterations in cardiac cycle patterns. The Grad-CAM analysis demonstrates that the model’s decision logic is consistent with these physiological mechanisms—the CNN-BiLSTM learns medically meaningful patterns rather than functioning as a black box. The model appropriately attends to R-peaks for normal state discrimination, incorporates beat-to-beat variability features for slight fatigue detection, and analyzes full waveform dynamics for fatigued state identification.

### 3.7. Robustness Validation with Alternative Data Splitting Strategies

To address potential temporal correlation bias in segment-level random splitting, we evaluated the model using session-wise and subject-wise data splitting strategies.

Session-wise splitting allocated complete 10 min recording sessions (each containing 20 consecutive 30 s segments) to training or test sets without overlap. Based on approximately 26–27 independent sessions, roughly 21 sessions were assigned to training and 5–6 sessions to testing. This approach eliminates temporal correlation bias while allowing within-subject learning across different time points.

Subject-wise splitting assigned all data from certain participants exclusively to either the training or test sets. We employed leave-2-subjects-out validation, randomly selecting 2–3 subjects for testing and using the remaining 9–10 subjects for training. This procedure was repeated 5 times with different random subject combinations, providing the most stringent cross-subject generalization assessment.

Table 8 presents comprehensive performance comparisons. Session-wise evaluation achieved 79.5% accuracy (F1: 0.788), a 3.5% reduction from segment-level (83.0%). This modest decrease reflects evaluation on unseen temporal contexts with natural physiological variations (circadian rhythms, post-activity recovery) while maintaining individual cardiac characteristics, indicating that the model has learned temporally generalizable fatigue patterns. Subject-wise evaluation achieved 74.1% accuracy (F1: 0.728), an 8.9% reduction from the segment-level. This larger decrease reflects substantial inter-individual variations in baseline heart rate and HRV ranges, compounded by the small test set size, where individual physiological differences become more pronounced.

Critically, both approaches substantially exceed all baseline methods: session-wise shows +15.2% improvement and subject-wise shows +9.8% improvement over the best traditional ML method (SVM: 64.3%). Per-class analysis reveals that non-fatigue and fatigued states maintain relatively stable performance across splitting methods (non-fatigue: 84.5%→82.9%→78.3%; fatigued: 84.2%→80.4%→74.6%), while slight fatigue shows higher sensitivity (80.7%→77.1%→70.6%), reflecting the inherent ambiguity of this intermediate state.

These rigorous evaluations confirm that the CNN-BiLSTM model learns meaningful and generalizable fatigue representations rather than exploiting dataset-specific temporal correlations.

### 3.8. Sensitivity Analysis of ECG Segment Duration

To validate the choice of 30 s ECG segments, we conducted sensitivity analysis testing seven different durations: 10 s, 15 s, 20 s, 30 s, 60 s, 90 s, and 120 s. For each duration, we re-segmented the original recordings, maintained identical train-test splits, and applied the same CNN-BiLSTM architecture with consistent hyperparameters. Results (Table 9) reveal an optimal performance range of 20–60 s. Classification accuracy increases from 73.5% (10 s) to 81.1% (20 s), reaches 83.3% at 30 s, and plateaus at 83.5% (60 s). Segments longer than 60 s show marginal improvements (+0.1–0.2%) that do not justify the doubled response latency for real-time monitoring. The 30 s duration provides an optimal balance between classification performance and practical responsiveness. This duration aligns with ultra-short-term HRV analysis standards and contains approximately 30–50 cardiac cycles, providing adequate statistical reliability for capturing autonomic nervous system variations associated with fatigue states. Segments shorter than 20 s contain insufficient cycles for reliable HRV analysis, while segments longer than 60 s compromise real-time monitoring capability without substantial accuracy gains. The corresponding performance of the model under different segment durations is shown in the table below.

## 4. Discussion

### 4.1. Advantages and Limitations

Our CNN-BiLSTM model achieved 83.3% accuracy in three-class fatigue classification using ECG signals from natural daily environments. Compared to existing studies focusing on binary classification in controlled settings (Aryal et al.: 82% with SVM; Huang et al.: 75.5% with KNN; Umer et al.: 95% with extreme physical tasks), our approach demonstrates key advantages: comparable performance on more challenging three-class classification and data collection in completely natural environments without stimulating tasks. The session-wise (79.5%) and subject-wise (74.1%) validation results still outperform traditional methods and show reasonable generalization.

ECG signal quality in natural environments presents inherent challenges. Approximately 12% of segments were rejected due to noise or motion artifacts (SNR < 15 dB), with morning recordings showing higher quality than evening recordings. The subjective Borg RPE scale labeling also introduces uncertainty, as participants sometimes struggled to distinguish between slight fatigue and fatigued states, partially explaining the lower accuracy for the slight fatigue class. Hyperparameter selection significantly impacts performance. Systematic grid search and sensitivity analysis revealed the optimal configuration (kernel: 150, filters: 32, BiLSTM units: 50, learning rate: 0.0001, dropout: 0.5), with performance variation ranging from 76.8% to 83.3% across combinations.

Several limitations should be acknowledged: a relatively small and homogeneous sample (12 participants), short monitoring period (two weeks), lack of external validation, exclusive focus on ECG signals, and no distinction between physical and mental fatigue components.

### 4.2. Future Work

Comprehensive validation across diverse populations is essential for clinical deployment. Future studies should include multiple age groups, various occupational categories, different activity levels, and participants with varying health conditions, with larger sample sizes (50–100 per group) to establish robust benchmarks. Extended longitudinal studies (months to years) are needed to investigate chronic fatigue development, track individual fatigue trajectories, and assess long-term model stability. Such studies would reveal how daily fatigue accumulates into chronic states and enable early intervention strategies. External validation through multi-center studies with independent datasets and cross-device validation across different wearable sensors is critical for establishing clinical credibility and ensuring hardware-independent performance. Future research should explore multimodal sensor fusion approaches. We are developing improved devices addressing data quality challenges in PPG and accelerometry. Integrating ECG, PPG, and motion data could capture complementary fatigue information and improve system robustness. Real-world deployment studies with at-risk populations (construction workers, drivers, healthcare professionals) would validate practical utility and identify implementation challenges. Development of personalized models adapting to individual characteristics could further enhance detection sensitivity.

## 5. Conclusions

In this study, we have designed a fatigue detection system that comprises a wearable device and a companion app, enabling the display and data storage of ECG signals. We conducted experiments involving 12 subjects, collecting ECG data in entirely natural, unstimulated living environments. Subsequently, we manually extracted 11 HRV-related features to use as inputs for developing three machine learning models aimed at classifying fatigue states.

Recognizing the time-consuming nature of manual feature extraction and the need for enhanced fatigue classification, we introduced a CNN-BiLSTM deep learning model. The results suggest that this proposed method can be regarded as a stable and dependable approach to monitor physical fatigue, making it suitable for detecting fatigue states in everyday life scenarios.

For future research, it would be valuable to explore the integration of contactless information for daily fatigue monitoring and expand the scope of subjects involved in the study.

## Figures and Tables

**Table 1:** The summer of key characteristics of prior work.

Authors	Environment	Methods	Best Performance
Aryal et al.	laboratory environment	Boosted trees(best)	82% on binary classification
Kushan et al.	Laboratory environment	SVM(best)	83.7% on binary classification
Umer et al.	Laboratory environment	Bagged tree(best)	98.5% on binary classification
Karvekar et al.	Laboratory environment	SVM(best)	61% on binary classification
Huang et al. Our work	Laboratory environment Natural environments without any additional stimulating tasks non-stimulating tasks fatigue experiment	KNN(best) Machine learning models and a convergent deep learning model	75.5% on binary classification 83.3% on three-class classification

**Table 2:** The structure of the first block.

Number of Layers in the First Block	Layer Type	Size
1	Input	
2	Convolution layer	Number of filters = 32, kernel size = 150, Stride = 6
3	ReLU	
4	Dropout Layer	Probability = 0.5
5	Max-pooling layer	Pooling size = 5

**Table 3:** The structure of the second block.

Number of Layers in the Second Block	Layer Type	Size
1	BiLSTM1	Size = 50, Stride = 20
2	BiLSTM2	Size = 50, Stride = 20
3	FC layer	
4	Dropout layer	Probability = 0.5
5	Softmax layer	Number of classes = 3

**Table 4:** The hyper-parameters of the proposed model.

Hyper-Parameters of the Proposed Model	Value/Type
Batch size	120
Learning rate	0.0001
Dropout	0.5
Number of epochs	2000
Pooling layer	Max pooling
Activation function	ReLU
Window size	20 Sec
Optimizer	Adam
Loss Function	Cross Entropy

**Table 5:** The results of machine learning models.

Model	Accuracy	Precision	F1 Score
Logistic Regression	0.602	0.627	0.591
SVM	0.643	0.670	0.629
Random Forest	0.574	0.591	0.558

**Table 6:** Comprehensive performance.

Method Category	Method	Accuracy	F1-Score	Precision	Recall
Simple Baselines					
	Single-Feature Threshold (RMSSD)	54.8%	0.532	0.541	0.524
	Rule-Based Classification	57.2%	0.556	0.563	0.549
	Weighted Scoring System	59.6%	0.581	0.589	0.573
Traditional ML					
	Support Vector Machine (SVM)	64.3%	0.628	0.641	0.616
	Random Forest (RF)	57.4%	0.561	0.573	0.550
	Logistic Regression	60.7%	0.594	0.605	0.584
Deep Learning					
	CNN-only	72.05%	0.708	0.721	0.696
	BiLSTM-only	75.86%	0.747	0.758	0.737
	CNN-BiLSTM (Proposed)	83.3%	0.825	0.834	0.816

**Table 7:** Statistical Test Results.

Comparison	Model 1 Accuracy	Model 2 Accuracy	Δ%	*p*-Value
C-BL vs. Random Forest	83.3%	57.4%	25.9%	0.0002
C-BL vs. Log Reg	83.3%	60.2%	23.1%	0.0004
C-BL vs. SVM	83.3%	64.3%	19.0%	0.0009
C-BL vs. CNN-only	83.3%	72.05%	11.25%	0.0031
C-BL vs. BiLSTM-only	83.3%	75.86%	7.44%	0.0083

**Table 8:** Performance comparison across data splitting strategies.

Metric	Segment-Level (Original)	Session-Wise	Subject-Wise	Baseline (SVM)
Overall Accuracy	83.0%	79.5%	74.1%	64.3%
Overall F1-Score	0.820	0.788	0.728	0.629
Std Deviation (±)	0.012	0.024	0.038	0.018
Non-fatigue F1	0.845	0.829	0.783	0.658
Slight fatigue F1	0.807	0.771	0.706	0.592
Fatigued F1	0.842	0.804	0.746	0.637
vs. Segment-level	-	−3.5%	−8.9%	−18.7%
vs. Baseline (SVM)	+18.7%	+15.2%	+9.8%	-

**Table 9:** Model performance across different ECG segment durations.

Segment Duration	Three-Class Accuracy	F1-Score	Precision	Recall
10 s	75.3% ± 2.1%	0.74	0.75	0.74
15 s	78.2% ± 1.8%	0.77	0.78	0.77
20 s	81.1% ± 1.5%	0.80	0.81	0.80
30 s	83.0% ± 1.2%	0.82	0.83	0.82
60 s	83.5% ± 1.4%	0.83	0.84	0.83
90 s	83.4% ± 1.6%	0.82	0.83	0.82
120 s	83.2% ± 1.8%	0.82	0.83	0.81

## Data Availability

The original contributions presented in this study are included in the article. Further inquiries can be directed to the corresponding author.

## Appendix A

**Table A1:** The summer of HRV features extracted.

Domain	Features	Description	Calculation Formulas
Time Domain	Mean SDNN SDSD RMSSD CVCD PNN50	Mean value of RR The standard deviation of all RR intervals The standard deviation The root mean square of successive differences The ratio of RMSSD to mean HR The proportion of the difference between adjacent intervals of more than 50 ms	$meanRR=\frac{1}{N}\sum_{i=1}^{N}\;RR_{i}$ (A1) $SDNN=\sqrt{\frac{1}{N}\sum_{i=1}^{N}\;{\left(RR_{i}-\mathrm{meanRR}\right)}^{2}}$ (A2) $SDSD=\sqrt{\frac{1}{N}\sum_{i=1}^{N}\;{\left[\left(RR_{i}-RR_{i+1}\right)-\left(\mathrm{meanRR}-RR_{i+1}\right)\right]}^{2}}\;$ (A3) $RMSSD=\sqrt{\frac{1}{N-1}\sum_{i-1}^{N-1}\;{\left(RR_{i+1}-RR_{i}\right)}^{2}}$ (A4) $CVCD=\frac{RMSSD}{\mathrm{meanRR}}\times 100\%$ (A5) $PNN50=\frac{NN50}{N-1}\times 100\%$ (A6)
Frequency Domain	$VLF_{\mathrm{percent}\;}$ $LF_{\mathrm{percent}\;}$ $HF_{\mathrm{percent}}$	The percent of very low frequency (VLF, 0.003–0.04 Hz) The percent of low frequency (LF, 0.04–0.15 Hz) The percent of high frequency (HF, 0.15–0.4 Hz)	$VLF_{\mathrm{percent}}=\frac{VLF\;\mathrm{power}}{\mathrm{Total}\;\mathrm{power}}\times 100\%$ (A7) $LF_{\mathrm{percent}}=\frac{LF\;\mathrm{power}}{\mathrm{Total}\;\mathrm{power}}\times 100\%$ (A8) $HF_{\mathrm{percent}}=\frac{HF\;\mathrm{power}}{\mathrm{Total}\;\mathrm{power}}\times 100\%$ (A9)
Nonlinear Domain	*Shannon Entropy* *RCMSE* [47]	The given discrete probability distribution Refine Composite Multiscale Entropy is using different scales of local matching patterns to compute the regularity of the signal.	$\mathrm{Shannon}\;\mathrm{Entropy}=-\sum_{i=1}^{N}\;p_{i}\log\left(p_{i}\right)$ (A10) $\mathrm{RCMSE}\left(x,\tau ,m,r\right)=-\ln\left(\frac{\sum_{k=1}^{\tau }\;n_{k,\tau }^{m+1}}{\sum_{k=1}^{\tau }\;n_{k,\tau }^{m}}\right)$ (A11)

## References

[1] Rahimian Aghdam S., Alizadeh S.S., Rasoulzadeh Y., Safaiyan A. (2019). Fatigue Assessment Scales: A Comprehensive Literature Review. Int. J. Occup. Saf. Ergon..

[2] Aryal A., Ghahramani A., Becerik-Gerber B. (2017). Monitoring Fatigue in Construction Workers Using Physiological Measurements. Autom. Constr..

[3] Health and Safety Executive (2006). Managing Shiftwork-Health and Safety Guidance.

[4] Allik A., Pilt K., Viigimäe M., Fridolin I., Jervan G. (2022). A Novel Physical Fatigue Assessment Method Utilizing Heart Rate Variability and Pulse Arrival Time towards Personalized Feedback with Wearable Sensors. Sensors.

[5] Aaronson L.S., Teel C.S., Cassmeyer V., Neuberger G.B., Pallikkathayil L., Pierce J., Press A.N., Williams P.D., Wingate A. (1999). Defining and Measuring Fatigue. Image J. Nurs. Scholarsh..

[6] Kuschan J., Krüger J. (2021). Fatigue Recognition in Overhead Assembly Based on a Soft Robotic Exosuit for Worker Assistance. Comput. Ind. Eng..

[7] Wang Y., Huang Y., Gu B., Cao S., Fang D. (2023). Identifying Mental Fatigue of Construction Workers Using EEG and Deep Learning. Autom. Constr..

[8] Umer W., Li H., Yantao Y., Antwi-Afari M.F., Anwer S., Luo X. (2020). Physical Exertion Modeling for Construction Tasks Using Combined Cardiorespiratory and Thermoregulatory Measures. Autom. Constr..

[9] Li J., Zhu J., Guan C. (2024). Assessing Illumination Fatigue in Tunnel Workers Through Eye-Tracking Technology: A Laboratory Study. Appl. Ergon..

[10] Hopstaken J.F., Van Der Linden D., Bakker A.B., Kompier M.A. (2015). A Multifaceted Investigation of the Link Between Mental Fatigue and Task Disengagement. Psychophysiology.

[11] Shortz A.E., Franke M., Kilic E.S., Peres S.C., Mehta R.K. (2017). Evaluation of Offshore Shiftwork Using Heart Rate Variability. Proceedings of the Human Factors and Ergonomics Society Annual Meeting.

[12] Bouneffouf D., Rish I. (2019). A Survey on Practical Applications of Multi-Armed and Contextual Bandits. arXiv.

[13] Yung M., Lang A.E., Stobart J., Kociolek A.M., Milosavljevic S., Trask C. (2017). The Combined Fatigue Effects of Sequential Exposure to Seated Whole Body Vibration and Physical, Mental, or Concurrent Work Demands. PLoS ONE.

[14] Marcora S.M., Staiano W., Manning V. (2009). Mental Fatigue Impairs Physical Performance in Humans. J. Appl. Physiol..

[15] Smith M.R., Marcora S.M., Coutts A.J. (2015). Mental Fatigue Impairs Intermittent Running Performance. Med. Sci. Sports Exerc..

[16] Naranjo-Flores A.A., Ramírez-Cárdenas E. (2014). Human Factors and Ergonomics for Lean Manufacturing Applications. Lean Manufacturing in the Developing World.

[17] Montenegro J.L.Z., da Costa C.A., da Rosa Righi R. (2019). Survey of Conversational Agents in Health. Expert Syst. Appl..

[18] Kajimoto O. (2008). Development of a Method of Evaluation of Fatigue and Its Economic Impacts. Fatigue Science for Human Health.

[19] Liu H., Yu Z., Chen C., Hong R., Jin K., Yang C. (2018). Visualization and Bibliometric Analysis of Research Trends on Human Fatigue Assessment. J. Med. Syst..

[20] Dontoh A., Ivey S., Sirbaugh L., Aboah A. (2025). A Review Paper of the Effects of Distinct Modalities and ML Techniques to Distracted Driving Detection. arXiv.

[21] Coetzer R., Hancke G. Driver Fatigue Detection: A Survey. Proceedings of the AFRICON 2009.

[22] Li K., Gong Y., Ren Z. (2020). A Fatigue Driving Detection Algorithm Based on Facial Multi-Feature Fusion. IEEE Access.

[23] Escobar-Linero E., Domínguez-Morales M., Sevillano J.L. (2022). Worker’s Physical Fatigue Classification Using Neural Networks. Expert Syst. Appl..

[24] Ricci J.A., Chee E., Lorandeau A.L., Berger J. (2007). Fatigue in the US Workforce: Prevalence and Implications for Lost Productive Work Time. J. Occup. Environ. Med..

[25] Zhou M., Zhang X. (2019). Online Social Networking and Subjective Well-Being: Mediating Effects of Envy and Fatigue. Comput. Educ..

[26] Bendak S., Rashid H.S. (2020). Fatigue in Aviation: A Systematic Review of the Literature. Int. J. Ind. Ergon..

[27] Wang H., Wu C., Li T., He Y., Chen P., Bezerianos A. (2019). Driving Fatigue Classification Based on Fusion Entropy Analysis Combining EOG and EEG. IEEE Access.

[28] Zhao L., Li M., He Z., Ye S., Qin H., Zhu X., Dai Z. (2022). Data-Driven Learning Fatigue Detection System: A Multimodal Fusion Approach of ECG (Electrocardiogram) and Video Signals. Measurement.

[29] Anwer S., Li H., Umer W., Antwi-Afari M.F., Mehmood I., Yu Y., Haas C., Wong A.Y.L. (2023). Identification and Classification of Physical Fatigue in Construction Workers Using Linear and Nonlinear Heart Rate Variability Measurements. J. Constr. Eng. Manag..

[30] Huang S., Li J., Zhang P., Zhang W. (2018). Detection of Mental Fatigue State with Wearable ECG Devices. Int. J. Med. Inform..

[31] Anwer S., Li H., Antwi-Afari M.F., Umer W., Wong A.Y. (2020). Cardiorespiratory and Thermoregulatory Parameters Are Good Surrogates for Measuring Physical Fatigue During a Simulated Construction Task. Int. J. Environ. Res. Public Health.

[32] Lee W., Lin K.-Y., Seto E., Migliaccio G.C. (2017). Wearable Sensors for Monitoring On-Duty and Off-Duty Worker Physiological Status and Activities in Construction. Autom. Constr..

[33] Xiang T. (2019). Research on the Association of Construction Workers’ Fatigue and Unsafe Behavior Based on Physiological Measurement. Master’s Thesis.

[34] Maman Z.S., Yazdi M.A.A., Cavuoto L.A., Megahed F.M. (2017). A Data-Driven Approach to Modeling Physical Fatigue in the Workplace Using Wearable Sensors. Appl. Ergon..

[35] Lamooki S.R., Kang J., Cavuoto L.A., Megahed F.M., Jones-Farmer L.A. Challenges and Opportunities for Statistical Monitoring of Gait Cycle Acceleration Observed from IMU Data for Fatigue Detection. Proceedings of the 2020 8th IEEE RAS/EMBS International Conference for Biomedical Robotics and Biomechatronics (BioRob).

[36] Karvekar S., Abdollahi M., Rashedi E. (2019). A Data-Driven Model to Identify Fatigue Level Based on the Motion Data from a Smartphone. Bioengineering.

[37] Othmani A., Sabri A.Q.M., Aslan S., Chaieb F., Rameh H., Alfred R., Cohen D. (2023). EEG-Based Neural Networks Approaches for Fatigue and Drowsiness Detection: A Survey. Neurocomputing.

[38] Adhikari S., Choudhury N., Bhattacharya S., Deb N., Das D., Ghosh R., Phadikar S., Ghaderpour E. (2025). Analysis of Frequency Domain Features for the Classification of Evoked Emotions Using EEG Signals. Exp. Brain Res..

[39] Tuncer T., Dogan S., Subasi A. (2021). EEG-Based Driving Fatigue Detection Using Multilevel Feature Extraction and Iterative Hybrid Feature Selection. Biomed. Signal Process. Control.

[40] Anwer S., Li H., Antwi-Afari M.F., Umer W., Wong A.Y.L. (2021). Evaluation of Physiological Metrics as Real-Time Measurement of Physical Fatigue in Construction Workers: State-of-the-Art Review. J. Constr. Eng. Manag..

[41] Kiyono K., Hayano J., Watanabe E., Yamamoto Y. (2017). Heart Rate Variability (HRV) and Sympathetic Nerve Activity. Clinical Assessment of the Autonomic Nervous System.

[42] Buendia R., Forcolin F., Karlsson J., Arne Sjöqvist B., Anund A., Candefjord S. (2019). Deriving Heart Rate Variability Indices from Cardiac Monitoring—An Indicator of Driver Sleepiness. Traffic Inj. Prev..

[43] Al-Libawy H., Al-Ataby A., Al-Nuaimy W., Al-Taee M.A. HRV-Based Operator Fatigue Analysis and Classification Using Wearable Sensors. Proceedings of the 2016 13th International Multi-Conference on Systems, Signals & Devices (SSD).

[44] Pan J., Tompkins W. (2007). A Real-Time QRS Detection Algorithm. IEEE Trans. Biomed. Eng..

[45] Yu Y., Rashidi M., Dorafshan S., Samali B., Farsangi E.N., Yi S., Ding Z. (2025). Ground Penetrating Radar-Based Automated Defect Identification of Bridge Decks: A Hybrid Approach. J. Civ. Struct. Health Monit..

[46] LeCun Y., Bengio Y., Hinton G. (2015). Deep Learning. Nature.

[47] Wang S.-H., Li H.-T., Chang E.-J., Wu A.-Y.A. Entropy-Assisted Emotion Recognition of Valence and Arousal Using XGBoost Classifier. Proceedings of the IFIP International Conference on Artificial Intelligence Applications and Innovations.

